# Metabolic engineering of yeasts by heterologous enzyme production for degradation of cellulose and hemicellulose from biomass: a perspective

**DOI:** 10.3389/fmicb.2014.00174

**Published:** 2014-04-22

**Authors:** William Kricka, James Fitzpatrick, Ursula Bond

**Affiliations:** School of Genetics and Microbiology, Department of Microbiology, Trinity College DublinDublin, Ireland

**Keywords:** recombinant yeasts, cellulases, xylose-utilizing enzymes

## Abstract

This review focuses on current approaches to metabolic engineering of ethanologenic yeast species for the production of bioethanol from complex lignocellulose biomass sources. The experimental strategies for the degradation of the cellulose and xylose-components of lignocellulose are reviewed. Limitations to the current approaches are discussed and novel solutions proposed.

## Introduction

With dwindling fossil fuel resources and the necessity to combat climate change by reducing greenhouse gas emissions, there is a growing need to identify alternative environmentally sustainable energy sources. One potential green energy source is that derived from biomass, which can be converted to useable energy such as biofuels. The two most common examples of biofuels are bio-ethanol and bio-diesel. Biofuels are considered the cleanest liquid fuel alternative to fossil fuel and it is estimated that replacing fossils fuels with biofuels could decrease CO_2_ emissions by 60–90% (Hasunuma and Kondo, 2012). Currently, over 100 billion liters of biofuels are produced annually, yet this accounts for only a fraction (2.7%) of total energy used in transportation. Bioethanol production reached 86 billion liters in 2010, with the United States and Brazil as the world's top producers, accounting together for 90% of global production (Buijs et al., 2013).

## Generations of bioethanol

Bioethanol production has been classified into different generations based on biotechnology developments and also in terms of the feedstock used. First generation bioethanol is produced from either corn or sugarcane. The released sugars, glucose and sucrose, respectively, are readily fermentable into ethanol by microorganisms such as the yeast *Saccharomyces cerevisiae* (Buijs et al., 2013). A disadvantage to using these substrates as energy sources is the fact that they are food crops and hence controversy regarding the ethics of exploiting “food for fuel” surrounds the established bioethanol industry. Currently over 90% of the world's bioethanol is produced from food crops, however governmental directives are setting exacting targets on the limitation of generating renewable energy from food-based crops. The US Renewable Fuel Standards mandate (RFS; US Energy Policy Act (EPAct) 2005) requires that 44% (16 billion gallons) of renewable-fuel to be blended into gasoline by 2022 be derived from non-food cellulosic biomass. Current negotiations at the European Council aim to limit the amount of biofuels from food-based crops that can be counted toward the 10% target for renewable energy in the transport sector by 2020 (EU Renewable Energy Directive (RED; 2009/28/EC).

Second generation biofuels are derived from more complex non-food based biomass, which can be grouped into roughly four categories, namely, wood residues, municipal solid waste, agricultural waste, and dedicated energy crops. The most abundant renewable form of biomass is lignocellulose comprising between 50 and 90% of all plant matter. The global production of plant biomass amounts to approximately 2 × 10^11^ Mt per annum of which 8–20 × 10^9^Mt is potentially accessible for processing. Lignocellulose is composed of three major components, cellulose, hemicellulose, and lignin. The relative amount of each component varies in different plant types, with an average composition of cellulose 30–50%, hemicellulose 20–30%, and lignin 15–25%.

Cellulose is the most abundant polysaccharide on earth (Chandel and Singh, 2011). In its simplest chemical form, cellulose is a β-glucan linear polymer of D-glucose linked by β-1,4-glycosidic bonds. The basic repeating subunit is cellobiose, consisting of two glucose molecules. At the macroscopic level, cellulose exists as two distinct forms, tightly packed crystalline and non-organized amorphous regions. The amorphous regions of cellulose are believed to result from surface shaving caused by natural erosion. Both the amorphous and crystalline forms are made up of cellulosic fibers comprising microfibrils, which are composed of approximately 30 β-glucan chains. The highly accessible amorphous regions account for approximately 1% of the structure of cellulose (Ruel et al., 2012).

Hemicellulose is the second most abundant polysaccharide within lignocellulose (Peng et al., 2012). It is a highly branched heteropolymer composed of pentoses and hexoses such as xylose, arabinose, mannose, glucose, and galactose as well as sugar acids. The composition of hemicellulose is variable in nature and depends upon the plant source, however the most abundant component of hemicellulose is generally xylose.

The third component of lignocellulose is lignin, a polymer of three aromatic alcohols, coniferyl, p-coumaryl, and sinapyl. Lignin links both hemicelluloses and cellulose together forming a physical barrier in the plant cell wall. Lignin is recalcitrant to degradation and is resistant to most microbial attacks and oxidative stress (Dashtban et al., 2009). Unlike cellulose and hemicellulose, hydrolysis of lignin does not generate fermentable sugars. Furthermore, phenolic compounds produced during lignin hydrolysis actively inhibit fermentation.

Biomass is not readily fermentable and expensive pre-treatments, both physical (milling and steam explosion) and chemical (acid and alkaline hydrolysis), are required to increase access to the sugars within the biomass. The sugars released from pre-treatment must be further hydrolyzed by enzymatic actions to yield fermentable glucose.

Several different approaches have been developed to generate bioethanol from biomass. One such process, called separate hydrolysis and fermentation (SHF), as its name suggests, requires a two-step process in which biomass hydrolysis and fermentation of released sugars are performed in separate reaction vessels. The main advantage of this method is that the two processes can be performed under their own individual optimal conditions. Hydrolysis of cellulose by enzymes, referred to as cellulases, is most efficient at temperatures between 50 and 60°C, whereas fermentation reactions are generally performed at 25–35°C, the optimum temperature range for yeast growth and metabolism. Cellulase enzymes are not found naturally in fermenting microorganisms and must be supplied *ex vivo*. A disadvantage of SHF is that glucose and cellobiose released by the action of cellulases inhibit the subsequent activity of enzymes.

To streamline production, an alternative process of simultaneous saccharification and fermentation (SSF) was devised. In this process the saccharification and fermentation are performed together in a single vessel and glucose released by the action of cellulases is immediately metabolized, thereby reducing enzyme inhibition. However, a disadvantage with SSF is the need to use a compromise temperature that is neither optimal for hydrolysis or fermentation.

The requirement for maximum efficiency to ensure economic viability of bioethanol production led to the development of consolidated bioprocessing (CBP) or third generation biofuels. In CBP, all steps are performed in the same reaction vessel by a single organism capable of both producing biomass hydrolyzing enzymes and fermenting the resultant sugars to ethanol. Thus, CBP avoids the need to supply exogenously produced cellulase enzymes.

## The cellulases

Cellulases belong to the O-glycoside hydrolases group of enzymes. There are three major classes of cellulases, endoglucanases (EG), cellobiohydrolases (CBH) or exoglucanases, and β-glucosidases (BGL). The concerted action of all three cellulases is required to efficiently convert cellulose into glucose. The generally accepted view is that cellulases act sequentially and synergistically. Endoglucanases randomly cleave the cellulose backbone at amorphous sites along the cellulose fiber. This leads to a rapid decrease in the degree of polymerization of the cellulose fiber and exposes new chain ends. Cellobiohydrolases act processively on reducing and non-reducing chain ends to release mainly cellobiose. β-Glucosidases hydrolyze the β-1,4 glycosidic bond of cellobiose and oligosaccharides to release glucose units.

Several novel classes of enzymes such as the copper-requiring polysaccharide monooxygenases, for example GH61, contribute to cellulose degradation by acting in synergy with the exo- and endoglucanases (Leggio et al., 2012; Žifčáková and Baldrian, 2012). Elastin-like proteins such as swollenin and other cellulase-enhancing proteins contribute to the hydrolysis of cellulose by increasing access of the cellulase enzymes to the cellulose chains ends (Kubicek, 2013; Nakatani et al., 2013). Cellulase enzymes are naturally produced by a variety of filamentous fungi of different genera such as *Trichoderma, Aspergillus, Talaromyces*, and several anaerobic bacteria. Two different cellulase systems, referred to as complexed and non-complexed have been described. Complexed cellulase systems are multi-enzyme complexes, referred to as the cellulosome, that remain tethered to the cell wall of cellulolytic bacteria (Lynd et al., 2002; Fontes and Gilbert, 2010). These cell wall tethered complexes are primarily encountered in anaerobic bacteria such as species of the *Clostridium* and *Ruminococcus* genera. Filamentous fungi and certain actinomycete bacteria such as *Cellulomonas* species use non-complexed cellulase systems. Non-complexed cellulases are secreted into the extracellular environment and are not attached to the cell surface. The most extensively studied cellulolytic organism is the filamentous fungus *Trichoderma reesei. T. reesei* synthesizes an array of cellulases, including at least five EGs, two CBHs, and two BGLs (Foreman et al., 2003; Martinez et al., 2008; Kubicek, 2013).

## Strategies for hydrolysis of cellulose and fermentation of released sugars to ethanol

While clearly capable of degrading cellulose, *T. reesei* is not an efficient fermenter of sugars to alcohol, a prerequisite for a CBP microorganism. *T. reesei* can generate up to 4.8 g/L ethanol from growth in glucose while *Aspergillus orzyae* can produce 24.4 g/L ethanol (Skory et al., 1997). Natural ethanologens such as *Zymomonas mobilis* or *Saccharomyces cerevisiae* can produce 130–200 g/L under the right environmental conditions. One major disadvantage with using mycelial fungi for ethanol production is the slow bioconversion rate compared to that observed in yeasts. Furthermore, filamentous fungi show intolerance to high concentrations of ethanol. An analysis of conversion of glucose to ethanol by 19 strains of *Aspergillus* revealed efficiencies of 21–98% after 6 days compared to 100% conversion by *S. cerevisiae* within 48 h (Skory et al., 1997).

To date, no naturally occurring microorganism capable of CBP at the desired efficiency for industrial bioethanol production has been identified. Therefore, researchers have pursued two strategies (native and recombinant) to generate the ideal microorganism for CBP. The native strategy focuses on modifying natural cellulolytic organisms to improve ethanol yields. Several approaches have been pursued, including directed evolution using error-prone Polymerase Chain Reaction-based mutagenesis of cellulase genes, adaptive evolution using natural selection to specific environmental conditions or rational protein design to improve the enzymatic activity of cellulases or to expand the physiological conditions at which the enzymes are active (Elkins et al., 2010; Voutilainen et al., 2010; Liang et al., 2011; Anbar et al., 2012; Gefen et al., 2012; Wang et al., 2012). Challenges still remain for these types of approaches in order to upscale to industrial fermentation conditions.

The recombinant strategy involves genetic engineering of native cellulase-producing species to improve ethanol yields or heterologous expression of cellulase genes in natural ethanologens. Ethanol yields in *A. niger* can be increased by expression of a pyruvate decarboxylase gene from *Z. mobilis* (Skory et al., 1997). Likewise, heterologous gene expression of pyruvate decarboxylase and alcohol dehydrogenase genes from *Z. mobilis* in the cellulolytic bacterium *C. cellulolyticum* was found to increase ethanol production by 53% (Guedon et al., 2002).

The vast majority of research into producing a CBP candidate has however followed the recombinant strategy of heterologous expression of cellulase genes in natural ethanologens. In order to achieve complete hydrolysis of cellulose, at least one copy of each of the three classes of cellulase genes must be expressed in the host cell. The most commonly used host for heterologous expression of cellulase genes for CBP is the baker's yeast, *S. cerevisiae* (Fujita et al., 2004; Den Haan et al., 2007a; Tsai et al., 2010; Wen et al., 2010; Yamada et al., 2011; Fan et al., 2012; Nakatani et al., 2013), although the three classes of cellulase genes have also been expressed in other *Saccharomyces* species such as *S. pastorianus* (Fitzpatrick et al., 2014) as well as in bacterial species such as *Escherichia coli* (Ryu and Karim, 2011) (Table 1). Since the native promoters of cellulase genes are repressed by glucose, strategies of using inducible or constitutive promoters of the host have been pursued. Inducible promoters such as *S. cerevisiae* GAL1 or CUP1 promoters are extremely efficient but require the addition of an inducer, galactose or copper, respectively. The requirement for such inducers can be expensive and often incompatible with fermentation conditions for ethanol production. Moreover, the GAL promoters are repressed in the presence of glucose and therefore not suited for industrial CBP. Partow et al. (2010) tested the performance of several constitutive and inducible promoters for heterologous gene expression in *S. cerevisiae*. Their findings indicated that the constitutive promoters TEF1 and PGK1 produced the most constant expression profiles. These promoters have been used for heterologous cellulase gene expression in *S. cerevisiae* (Den Haan et al., 2007b; Yamada et al., 2011), however no more than a 2-fold difference in expression was observed in genes driven by these two promoters, with TEF1 generating the highest levels (Fitzpatrick et al., 2014).

**Table 1:** **Ethanol production from PASC by recombinant yeast strains expressing cellulases**.

**Host strain**	**Cellulase enzyme**	**Cellulase system**	**PASC concentration (g/L)**	**Ethanol concentration (g/L)**	**Fermentation time (h)**	**References**
*S. cerevisiae*	*T. reesei* EGII, CBHII, *A. aculeatus* BGL1	Tethered	10	2.9	40	Fujita et al., 2004
*S. cerevisiae*	*T. reesei* EGI, *S. fibuligera* bgl	Secretion	10	1	192	Den Haan et al., 2007b
*S. cerevisiae*	*T. reesei* EGII, CBHII, *A. aculeatus* BGL1	Secretion	10	1.6	60	Yanase et al., 2010
*S. cerevisiae*	*T. reesei* EGII, CBHII, *A. aculeatus* BGL1	Tethered	10	2.1	60	Yanase et al., 2010
*S. cerevisiae*	*C. thermocellum* endoglucanase, *C. cellulolyticum* exoglucanase, *T. reesei* CBHII, *T. aurantiacus* BGLI	Minicellulosome	10	1.9	48	Tsai et al., 2010
*S. cerevisiae*	*T. reesei* EGII, CBHII, *A. aculeatus* BGLI, *C. thermocellum* miniscaffoldin	Minicellulosome	10	1.8	72	Wen et al., 2010
*S. cerevisiae*	*T. reesei* EGII, CBHII, *A. aculeatus* BGLI	Tethered	20	7.6	72	Yamada et al., 2011
*E. coli*	*C. cellulyticum* Cel5A, Cel9E, BGL	Tethered	10	3.6	60	Ryu and Karim, 2011
*S. cerevisiae*	*C. cellulyticum* celCCA, celCCE, Ccel_2454	Minicellulosome	10	1.1	96	Fan et al., 2012
*S. cerevisiae*	*T. reesei* EGII, CBHII, *A. aculeatus* BGLI *A. oryzae* AoelpI	Tethered	20	3.4	96	Nakatani et al., 2013
*S. pastorianus*	*T. reesei* EGI, CBHII, BGLI	Secretion	25	16.5	240	Fitzpatrick et al., 2014

Another strategy for increasing cellulase activity is to increase the gene copy number. Episomal plasmids have been extensively used to express cellulase genes (Fujita et al., 2004; Den Haan et al., 2007b; Tsai et al., 2010; Wen et al., 2010; Fan et al., 2012), however there is an issue with their genetic stability. Under non-selection, plasmids are lost in several generations and constant selection such as culturing in auxotrophic or antibiotic media must be applied. This set up is not suited to industrial production due to the cost of such selection reagents. The expression of *T. reesei* endoglucanase gene EGI on a high copy number 2 μ episomal vector (pRSH-series) was 50-fold greater than when expressed from an ARS/CEN vector (pGREG-series) (Fitzpatrick et al., 2014). A preferred solution is the integration of cellulase gene cassettes directly into the chromosome of the host microbe. Classic integration methods ensure stability of the genes, however, enzyme production is limited by gene copy number (Du Plessis et al., 2010; Yamada et al., 2013). Multi-copy integration of cellulase genes offers a solution. Yamada and co-workers constructed *S. cerevisiae* strains containing multiple-copies of cellulase genes integrated into the delta (δ) repeat sites of transposable elements (Tn) in the host chromosome, leading to increased ethanol yields (Yamada et al., 2010).

Defining the optimum ratio of the different classes of cellulases is also important to achieve maximum cellulose hydrolysis. The true optimum ratio of cellulases used by natural cellulolytic microorganisms is not fully known (Yamada et al., 2013) but it has been estimated that the total secreted protein of *T. reesei* under inducing conditions is 60% CBHI, 20% CBHII, 10% EG, and 1% BGL (Takashima et al., 1998). Hence, a 1:1:1 ratio of the three classes of enzymes may not yield an optimum hydrolysis synergy. Yamada and colleagues performed repeated rounds of integration at delta sites to generate an *S. cerevisiae* strain with 24 cellulase genes integrated into the genome in a ratio of 16:6:2 for *egl2, cbh2*, and *bgl*1, respectively (Tien-Yang et al., 2012). Cellulose degradation activity of this strain was lower than in a strain which had the cellulase genes *egl2, cbh2*, and *bgl*1 in a ratio of 13:6:1, respectively. Interestingly, a strain with *egl2, cbh2*, and *bgl*1 in a ratio of 5:9:6, respectively produced 1.3-fold less cellulose hydrolysis activity than a strain containing the genes in a 13:6:1 ratio, suggesting that the ratio of the cellulase enzymes as well as the copy number is crucial to ensure efficient cellulose hydrolysis.

**Figure 1:**
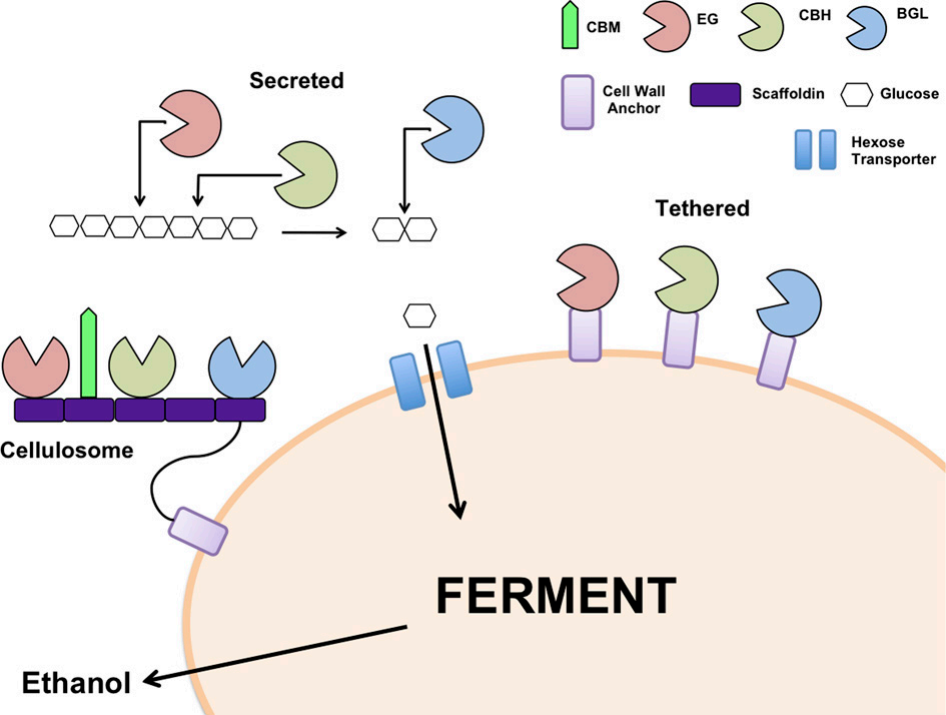
**Models for cellulase expression in yeast; heterologous cellulases can be secreted into the medium, tethered individually to the cell wall or assembled into a cellulosome**. EG: Endoglucanase, CBH: Cellobiohydrolase, BGL: β-glucosidase, CBM: Cellulose binding module.

The choice of cellulase system for integration into the recipient ethanologenic host is important to consider. The cellulosome from *C. thermocellum* has been reconstituted in *S. cerevisiae* (Wen et al., 2010) as have the secretory cellulases from several filamentous fungi including *A. aculeatus, A. oryzae, Saccharomycopsis fibuligera, Thermoascus aurantiacus, and T. reesei* (Table 1). Genes encoding the different classes of cellulases from a single species or from different fungal origins have been co-expressed in *S. cerevisiae* (Table 1). Secretion of the expressed cellulases has been achieved using the native secretory signals or host signals, for example, the α-mating type secretory signal. In some cases, the recombinant cellulases have been tethered to the cell surface (Table 1, Figure 1) using anchor proteins such as *S. cerevisiae* α-agglutinin (Yanase et al., 2010) or cell wall protein (Van Rooyen et al., 2005). Cell wall tethering leads to an effective increase in concentration of the cellulases. On the other hand, expressing cellulases so that the proteins are untethered facilitates the binding of the enzymes at multiple sites along the length of the cellulose chain. Interesting, very little difference in ethanol yields was observed in yeast strains in which the cellulases were secreted or cell wall tethered, indicating that one approach is not superior to the other (Yanase et al., 2010).

To date, haploid *S. cerevisiae* has been the host of choice for heterologous expression of cellulase genes, although a few studies have examined the expression of cellulase genes in other hosts. Yamada and colleagues constructed a diploid *S. cerevisiae* yeast strain with cellulase genes integrated at δ-integration sites. The diploid strain displayed 6-fold higher phosphoric acid swollen cellulose (PASC) degradation than the parental haploid (Yamada et al., 2011). Cellulases have also been expressed in the polyploid lager yeast *S. pastorianus* (Fitzpatrick et al., 2014). The latter study compared the expression of the three classes of cellulases from *T. reesei* in strains of *S. pastorianus* and *S. cerevisiae*. Enzymatic activity for all three classes of enzymes was up to 10-fold higher in *S. pastorianus* strains compared to *S. cerevisiae* strains. Thus for CBP, it will be important to explore the use of yeast species other than *S. cerevisiae* for cellulase production. This point is particularly important in light of the temperature difference between optimal growth conditions for *Saccharomyces* species (25–35°C) and for cellulases activity (50–60°C). Cellulase activity at 30°C is 3-fold less than at 50°C and therefore cellulose hydrolysis will always be compromised when *Saccharomyces* species are used as hosts. An organism that could bridge the gap between saccharification and fermenting temperatures is *Kluyveromyces* sp., which are more thermotolerant than *Saccharomyces* sp. (Fonseca et al., 2008). Expression of all three classes of cellulases in *K. marxianus* produced up to 43 g/L ethanol using the simple di-saccharide cellobiose as a sole carbohydrate source (Hong et al., 2007). The hydrolysis of more complex cellulose substrates such as PASC has yet to be tested with this recombinant host. One disadvantage with *K. marxianus* is that it is less ethanol tolerant than *S. cerevisiae*.

## The chicken and egg problem

Ultimately, a key requirement of a recombinant host species for CBP is the ability to utilize complex cellulose substrates such as lignocellulose biomass as the sole carbohydrate source. It remains unclear how the hydrolysis of cellulose by recombinant microbes can be initiated. The host must produce enzymes to degrade cellulose into glucose to ensure cell growth while at the same time, the cell must be growing in order to produce and secrete enzymes, thus the classic chicken and egg conundrum. Cellulose cannot be transported directly into the cell and must be hydrolyzed extracellularly. In order to produce the cellulases, the cells must be actively dividing to ensure transcription from inducible or constitutive promoters. It may be therefore necessary to supply a residual amount of fermentable sugars, such as glucose or sucrose to kick-start the process. We have previously shown that yeast cell growth can be maintained with as little as 0.5 g/L glucose, although the addition of sugars to CBP will ultimately add costs to the process. The approach taken by several groups is to use very high cell numbers to carry out fermentations of cellulose substrates. The idea here is that residual cellulases synthesized in the pre-fermentation starter cultures will be released from cells upon incubation with fresh medium containing the cellulose substrate. A disadvantage to this approach is that the use of high cell densities limits the continued growth of the culture, which inevitably will quickly enter stationary phase. Tethering the cellulases to the cell surface may solve this dilemma, as cellulases produced in pre-fermentation starter cultures may remain active once the cells are switched to the biomass fermentation process.

Taking the known problems associated with CBP into account, we favor an SHF *in situ* approach in which a pre-hydrolysis of the cellulose substrate is performed prior to fermentation. Pre-hydrolysis can be carried out with recombinant cellulases secreted into the spent medium in yeast starter cultures. This spent medium is then used in a pre-fermentation step to begin the process of hydrolysis of the cellulose substrate at the optimal temperature of 50–60°C to release glucose. The medium can then be cooled, replenished with essential nutrients and cellulase-expressing yeasts, and fermentations carried out at 25–30°C (Figure 3). Interestingly, the majority of industrial fermentation facilities used for the production of potable alcohols (beer, ales, lagers, and spirits) incorporate a pre-fermentation hydrolysis step at high temperatures (50–63°C) in order to release fermentable sugars (maltose, sucrose etc.) from complex carbohydrate substrates (starches) such as wheat and barley. Therefore, a SHF *in situ* approach could easily be incorporated into current fermentation processes. SHF *in situ* differs from SHF in eliminating the requirement for the addition of costly commercially produced cellulases.

The ultimate test of any CBP host candidate, whether generated by native or recombinant strategies, is the amount of ethanol the microorganism can produce from a cellulosic substrate. The most commonly used cellulosic substrate tested by research groups is phosphoric acid swollen cellulose (PASC). Table 1 summarizes the ethanol yields from fermentation of hydrolyzed PASC reported to date. Despite the myriad of approaches undertaken, ethanol levels are in the range of 7–16.5 g/L, far below what is achievable with more conventional complex carbohydrate substrates such as grains where yields of up to 200 g/L are commonplace. For industrial bioethanol production from cellulosic biomass, far greater yields are required. The low yields most likely reflect the slow bioconversion rate of cellulose-based substrates such as PASC. The results summarized in Table 1 highlight the need for further improvements in this process.

It should be noted that it is difficult to compare ethanol yields from the various studies due to variations in experimental parameters such as starting substrate concentrations, incubation times, and the density of cells used for fermentations. Furthermore, the unit of activity for cellulase hydrolysis differs widely. For comparative purposes, going forward, it would be useful for research groups to standardize the experimental conditions used for PASC hydrolysis and to adopt a standard definition of a unit of cellulase activity, for example, grams of ethanol produced per gram of theoretical glucose present in the cellulose substrate per hour (g ethanol g glucose^−1^ h ^−1^). Furthermore, while current studies almost exclusively use PASC as an experimental cellulose substrate, it will be essential to expand this analysis to the use of more complex lignocellulose substrates such as straw, spent grains, and grasses.

## Metabolism of xylose from hemicellulose for bioethanol production

The pentose sugar xylose is a major component of hemicellulose. Its abundance and relative ease of extraction from biomass by pre-treatments described above make it an attractive source of fermentable sugar for the production of bioethanol. In nature, xylose can be utilized by various yeast species and bacteria that are found associated directly or indirectly with lignocellulose. The mining and isolation of yeast from environments where xylose is likely to be an abundant natural sugar has developed greatly over recent years. Several xylose-utilizing yeast species such as *Spathaspora passalidarum* are indirectly associated with lignocellulose, via their symbiotic relationship with wood boring beetles such as *Odontotaenius disjunctus* (Hou, 2012) or wood roaches such as *Cryptocercus* sp. (Urbina et al., 2013). Additionally xylose-utilizing species including *Candida* sp., *Geotrichum* sp., *Sporopachydermis* sp., *Trichosporon* sp., *Pichia* sp., and *Sugiyamaella* sp. have been isolated from buffalo feces (Wanlapa et al., 2013) or from soil (Zhang et al., 2014). Although some success has been achieved using natural xylose fermenting species, their industrial relevance has yet to be demonstrated.

**Figure 2:**
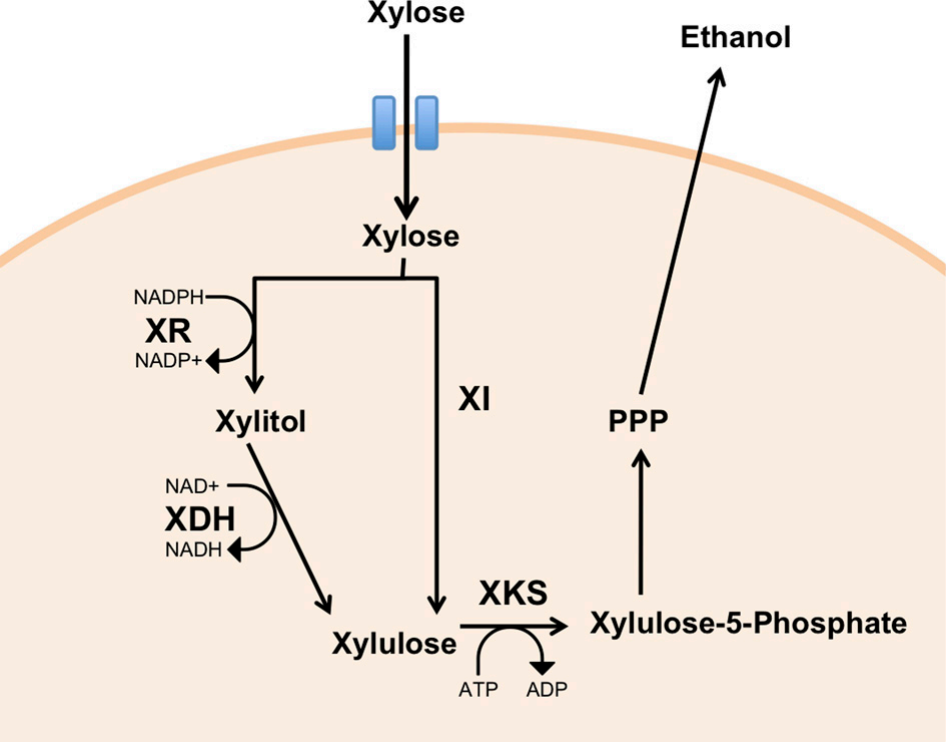
**Heterologous expression of xylose metabolic pathways in yeast**. Xylose can be metabolized by xylose reductase (XR) and xylitol dehydrogenase (XDH) (Pathway 1), or by xylose isomerase (XI) (Pathway 2) to yield xylulose. Phosphorylation of xylulose by xylulose kinase (XKS) yields xylulose-5-P, which can enter pentose phosphate pathway (PPP).

Two major xylose utilizing pathways have been identified. In xylose-fermenting fungi and yeasts, xylose utilization involves the action of two oxidoreductases, xylose reductase (XR) and xylitol dehydrogenase (XDH), each requiring the co-factors NADPH and NAD^+^, respectively in the forward reactions (Figure 2, pathway 1). Most bacterial species use an alternative pathway requiring just a single enzyme, xylose isomerase (Figure 2, pathway 2). The product of both pathways, xylulose, is phosphorylated by xylulose kinase, which can enter the Pentose Phosphate Pathway (PPP), thus generating intermediates of the glycolytic pathway.

While a few xylose utilizing *Saccharomyces* sp. have been identified (Wenger et al., 2010; Schwartz et al., 2012), in general, yeasts belonging to the *Saccharomyces stricto sensu* group cannot metabolize xylose despite harboring genes encoding putative xylose-utilizing enzymes. To facilitate the incorporation of xylose utilization into CBP, efforts have focussed on introducing xylose metabolic pathways from other species into natural ethanologenic *Saccharomyces* sp. (Karhumaa et al., 2007; Matsushika et al., 2009a,b; Fernandes and Murray, 2010; Bera et al., 2011; Hasunuma et al., 2011; Hector et al., 2011; Usher et al., 2011; Xiong et al., 2011; Cai et al., 2012; Fujitomi et al., 2012; Kim et al., 2012; Tien-Yang et al., 2012; De Figueiredo Vilela et al., 2013; Demeke et al., 2013; Hector et al., 2013; Ismail et al., 2013; Kato et al., 2013; Kim et al., 2013). This topic has been reviewed recently (Matsushika et al., 2009a; Fernandes and Murray, 2010; Cai et al., 2012) and so will not be extensively covered here. Instead, we summarize the various experimental approaches used, the heterologous genes that have been expressed and the ethanol yields generated (Table 2). As with the data on ethanol yields from cellulose (Table 1), it is difficult to compare results on ethanol yields from xylose, with different studies representing ethanol yields as ethanol (g/L^−1^), grams ethanol/g^−1^ xylose or % yield (Table 2). Despite the variety of approaches, the levels of ethanol produced are remarkably consistent ranging from 4 to 25 g/L, far below that which is achievable by native yeasts using carbohydrate sources such as glucose, maltose, or sucrose. These low yields reflect the poor uptake of xylose by *S. cerevisiae*, the species of choice for most of these studies and the slow metabolism of the sugar due to redox imbalances in the cell. Below we summarize some of the approaches that have been taken to improve these processes.

**Table 2:** **Ethanol production from xylose by recombinant yeast strains expressing xylose utilizing enzymes**.

**Host strain**	**XR**	**XDH**	**XI**	**XK**	**Ethanol (g/L)**	**Ethanol yield (g/g Xylose)**	**Ethanol yield (%)**	**References**
*S. cerevisiae* (4124)	*Pichia stipitis*	*Pichia stipitis*		*S. cerevisiae*			76.8	Bera et al., 2011
*S. cerevisiae* (4124)	*Neurospora crassa*	*Pichia stipitis*		*S. cerevisiae*			78.6	
*S. cerevisiae* (4124A)	*Candida parapsilosis*	*Pichia stipitis*		*S. cerevisiae*			34.2	
*S. cerevisiae* (YRH400)	*Pichia stipitis*	*Pichia stipitis*		*S. cerevisiae*	6.9		48.3	Hector et al., 2013
*S. cerevisiae* (YRH631)			*Prevotella ruminicola*	*Prevotella ruminicola*	4.1		68.6	
*S. cerevisiae* (YRH1114)			*Prevotella ruminicola*	*Prevotella ruminicola*	13.6		82.9	
*S. cerevisiae* (YY5A)	*Candida guilliermondii*	*Pichia stipitis*		*Pichia stipitis*	6.1	0.33		Tien-Yang et al., 2012
*S. cerevisiae* (MA-D4)	*Pichia stipitis*	*Pichia stipitis*		*S. cerevisiae*	14.1	0.35		Matsushika et al., 2009b
*S. cerevisiae* (MA-T4)	*Pichia stipitis*	*Pichia stipitis*		*S. cerevisiae*	15.7	0.35		
*S. cerevisiae* (Y7092)			*Piromyces* sp. *E2*	*S. cerevisiae*	5.3	0.35		Usher et al., 2011
*S. cerevisiae* (YPH499XU)	*Scheffersomyces stipitis*	*Scheffersomyces stipitis*		*S. cerevisiae*		0.24		Kato et al., 2013
*S. cerevisiae* (δ X-70)	*Scheffersomyces stipitis*	*Scheffersomyces stipitis*		*S. cerevisiae*		0.36		
*S. cerevisiae* (δ X-27)	*Scheffersomyces stipitis*	*Scheffersomyces stipitis*		*S. cerevisiae*		0.35		
*S. cerevisiae* (TMB 3057)	*Pichia stipitis*	*Pichia stipitis*		*S. cerevisiae*	13.3	0.33		Karhumaa et al., 2007
*S. cerevisiae* (TMB 3066)			*Piromyces* sp. *E2*	*S. cerevisiae*	7.3	0.43		
*S. cerevisiae* (TMB 3400)	*Pichia stipitis*	*Pichia stipitis*		*S. cerevisiae*	12.1	0.34		
*S. cerevisiae* (BY4741X)	*Scheffersomyces stipitis*	*Scheffersomyces stipitis*		*S. cerevisiae*	25.4	0.28		Fujitomi et al., 2012
*S. cerevisiae* (HDY.GUF5)			*Clostridium phytofermentans*	*S. cerevisiae*		0.23		Demeke et al., 2013

Pentose sugars can naturally be transported into *S. cerevisiae* by hijacking various hexose sugar transporters (Hxt7/Hxt5/Hxt4/Hxt2 and Gal2). The major problem encountered here is the greater affinity of these transporters for their natural substrate glucose. Therefore, in media containing both hexose and pentose sugars, such as that generated by pre-treatment of lignocellulose biomass, glucose is preferentially transported while xylose uptake is inhibited. Two distinct strategies, the overexpression of genes encoding native transporters from *S. cerevisiae* and the heterologous expression of genes encoding transporters from xylose utilizing species have been pursued. The genes encoding hexose transporters Hxt1, Hxt7, Hxt13, and Gal2 have been individually overexpressed in *S. cerevisiae*, with growth rates and xylose consumption varying between individual studies. Xylose transport was increased by the over expression of HXT1 and GAL2 but not by overexpression of HXT7 (Tanino et al., 2012). These studies revealed a trade off between transporter specificity and transporter efficiency, with transporters displaying high specificity (low Km) being less efficient at xylose transport (Young et al., 2012).

Based on these findings, the use of specific xylose transporters from xylose-utilizing species would seem like a more logical approach. Jaewoong et al. (2013) expressed genes encoding 6 xylose transporters from *Scheffersomyces stipitis* in *S. cerevisiae*, all of which demonstrated improved growth rates and ethanol production when grown solely on xylose, with the genes XUT7, RGT2, and SUT4 showing the most benefit. A directed mutagenesis of xylose transporters GXS1 and XUT3 from *Candida intermedia* and *S. stipites*, respectively led to improved growth rate on xylose (Young et al., 2012). As might be expected, the expression of heterologous xylose specific transporter genes shows improved xylose uptake in mixed hexose and pentose medium (Runquist et al., 2010), however, in some cases, strains where xylose consumption was improved, overall sugar consumption (glucose and xylose) was lower than that achieved by overexpression of native hexose transporters such as HXT7 and GAL2 (Young et al., 2011).

Identifying the best xylose transporters for heterologous expression also poses problems. A comparative study on the heterologous expression of genes encoding different sugar transporters in *S. cerevisiae* demonstrated that of an initial 23 transporters tested, only five conferred the ability to grow on xylose (Young et al., 2011). Likewise, an analysis of 18 genes encoding putative xylose transporters led to the cloning of just two xylose-specific transporters, XYP29 and AN25 from *Pichia stipitis* and *Neurospora crassa*, respectively (Du et al., 2010).

In addition to problems associated with xylose transport, xylose utilization in *S. cerevisiae* is hampered by bottlenecks in the metabolism of xylose through the PPP (Fiaux et al., 2003). Several studies have been conducted to improve the metabolic fluxes through the PPP, including the overexpression of genes encoding key enzymes in the pathway such as transaldolase (TAL1), transketolase (TKL1), ribose-5-phosphate isomerase (RK11), and ribulose 5-phosphate epimerase (RPE1) (Karhumaa et al., 2005). While over expression of these genes individually produced mixed outcomes, the over expression of all 4 PPP enzymes produced a 30-fold increase in growth rate on xylose, however ethanol yields only increased fractionally (Bera et al., 2011). Although over expression of TAL1 did not greatly improve ethanol production from synthetic xylose, improved ethanol yields were achieved when detoxified hemicellulosic hydrosylate was used as a sole carbohydrate source (Hasunuma et al., 2014).

Genome-wide expression analysis identified additional host genes required for optimum xylose utilization including the PPP-associated SOL3 and GND1 as well as non PPP-associated genes such GAL1, 7, and 10 (Wahlbom et al., 2003; Bengtsson et al., 2008). Deletion of YLR042C, MNI1, and RPA49, resulted in an improvement in growth rates on xylose (Bengtsson et al., 2008), while deletion of PHO13, ALP1, ISC1 RPL20B, BUD21, NQM1, TKL2 led to increased ethanol yields from xylose (Van Vleet et al., 2008; Usher et al., 2011).

It is clear that PPP involves complex regulation involving many gene products. Thus, major alterations in the PPP may be required to optimize xylose utilization by *S. cerevisiae*. While mutational and over expression approaches have helped to identify key genes regulating the PPP, the industrial relevance of any such modifications must be considered. Given the complexity of the system, an adaptive evolutionary approach involving continued rounds of cell conditioning on xylose might be a more beneficial approach to increasing xylose utilization in *S. cerevisiae*.

## Conclusions and perspectives

**Figure 3:**
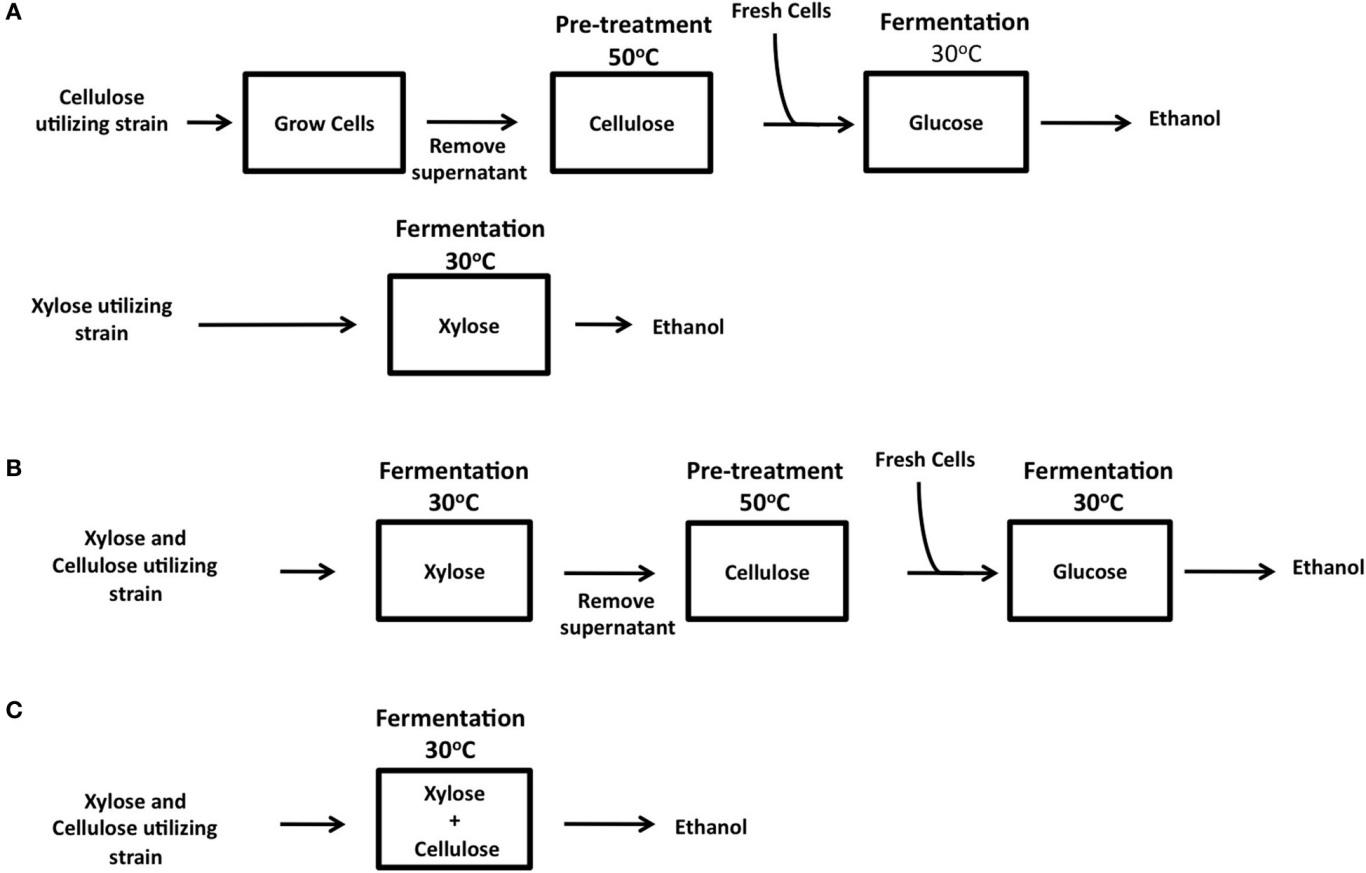
**Three possible methods for industrial lignocellulose conversion into bioethanol by SFH *in situ*. (A)** Parallel xylose and cellulose utilization using two different strains for separate xylose and cellulose fermentation, or in a combined process where both xylose and cellulose are utilized by a single strain in a sequential manner **(B)** or within one reaction **(C)**.

The ultimate goal of research into third generation biofuel is to create an organism capable of CBP. For the efficient exploitation of biomass as a source of bioenergy, it is obvious that both the cellulosic and hemicellulosic fractions of biomass must be used. The degradation of cellulose by cellulase expressing ethanologenic yeast strains is now well established, except for the caveat that to date only synthetic forms of cellulose have been tested (Table 1). The main stumbling block here is the poor enzyme activity of recombinant cellulases at fermentation conditions (30°C). Until activity can be increased at this low temperature, it is likely that there will be a need for an enzymatic pre-hydrolysis of biomass prior to fermentation, i.e., SHF *in situ* (Figure 3). To move the field forward, it is essential that the degradation of more complex biomass by engineered strains be tested, be it straw, grasses, or waste products such as spent grains. Progress on xylose fermentation has also been made, however both fields have been developing in parallel rather than merging into one. To date only cellobiose and xylose co-utilizing strains having been generated (Katahira et al., 2006; Ha et al., 2011; Aeling et al., 2012).

To achieve CBP, many criteria have to be satisfied. The ideal host should be capable of such metabolizing both cellulose and xylose, maintaining maximum heterologous expression of enzymes at the optimum ratio, be resistant to high ethanol concentrations and ideally be thermotolerant. Ideally, a single organism should possess all of these attributes, however the heterologous production of so many enzymes can reduce the fitness of the host cell (Tsai et al., 2010). Co-culturing yeast strains expressing individual cellulase or xylanases offers a easy way to vary the ratio of cellulase and xylanase enzymes by simply altering the number of cells expressing each enzyme in the fermentation. With the proposed SHF *in situ* approach, various scenarios can be envisioned. Cellulose and xylose can be fermented separately from pre-treated biomass (Figure 3A). Alternatively, yeast strains co-expressing xylose and cellulose utilizing genes together or co-cultures of strains expressing individual genes can be cultured on hydrolysates derived from pre-treatment of biomass. The available xylose and glucose in the hydrolysate can be used as energy sources to allow the growth of yeast and thus the production and secretion of cellulases into the medium (Figure 3B). The spent medium (supernatant) can then be used for enzymatic pre-hydrolysis of cellulose followed by a fermentation of released glucose. This step-wise process is compatible with current industrial fermentation processes. Ultimately, the most efficient CBP would require a single microorganism capable of fermentation of both xylose and cellulose in a single step at 30°C (Figure 3C). We eagerly await the development of this super strain!

In conclusion, every component involved in lignocellulosic bioethanol generation such as the host organism, hydrolyzing enzymes and even biomass substrate are now the focus of bioengineering research in the pursuit for greater efficiency to reduce production costs. Thus, the future of biofuel utilization is dependent on the economics of its production, which is itself reliant on the science used to generate it.

### Conflict of interest statement

The authors declare that the research was conducted in the absence of any commercial or financial relationships that could be construed as a potential conflict of interest.
